# Single-Molecule Technique Links Structural Fluctuations of Proteins to Brain Diseases

**DOI:** 10.1371/journal.pbio.1001338

**Published:** 2012-05-29

**Authors:** Janelle Weaver

**Affiliations:** Freelance Science Writer, Glenwood Springs, Colorado, United States of America

**Figure pbio-1001338-g001:**
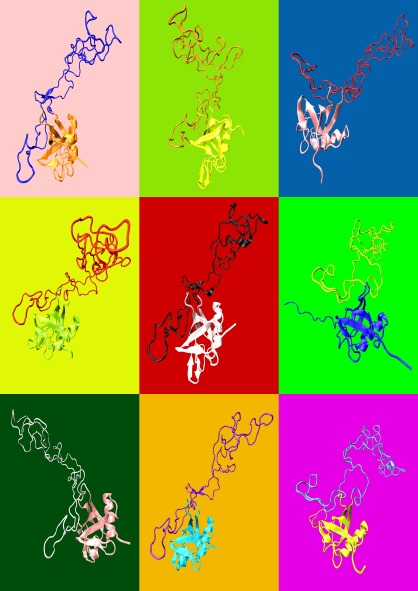
Conformational polymorphism of a neurotoxic protein. (Artistic rendering by Àngel Gómez-Sicilia and Abert Galera-Prat.)

[Fig pbio-1001338-g001]Diseases such as Alzheimer's, Parkinson's, and Huntington's are characterized by a dramatic loss of neurons and are believed to be caused by the accumulation of neurotoxic proteins either inside or outside of cells. Although distinct neurotoxic proteins are implicated in various neurodegenerative disorders, they are thought to undergo a structural change that triggers protein aggregation and a series of events resulting in cell death. However, established techniques have made it challenging for scientists to determine the key conformational change at the molecular level that leads to disease.

In this issue of *PLoS Biology*, a team led by Mariano Carrión-Vázquez at the Cajal Institute used a novel approach to unmistakably identify specific structural conformations of individual molecules, thereby revealing how these conformations relate to cellular dysfunction in a range of neurodegenerative diseases. The researchers found that extremely stable molecular conformations in various neurotoxic proteins are associated with protein aggregation. Moreover, their results suggest that a single drug may be able to target shared structural features in these proteins, which could help to prevent or treat multiple neurodegenerative disorders.

By modifying existing protocols for a high-resolution biophysical technique known as atomic force microscopy (AFM)-based single-molecule force spectroscopy (SMFS), Carrión-Vázquez and his team achieved an unprecedented level of data quality, which allowed them to carefully examine the mechanical stability of molecular conformations in neurotoxic proteins. Using this tool, they measured the forces necessary to unfold the proteins by attaching one end of the proteins to a sharp tip and the other end to a surface, which stretched the proteins when the surface was pulled away from the tip. To avoid data contamination that has plagued past experiments, they inserted the proteins of interest into protective carrier proteins.

Using AFM-based SMFS, the researchers found a higher frequency of stable conformations in fatal proteins implicated in Alzheimer's, Parkinson's, Huntington's, and prion diseases than in nontoxic proteins. These structures were even more abundant in mutated proteins that are linked to the early onset and increased severity of disease. The structural stability of a small fraction of these conformations was the highest ever reported for any protein.

Moreover, the robust conformations were associated with protein aggregation, and these structures formed less frequently when the noxious proteins were exposed to a neuroprotective peptide that inhibits the abnormal accumulation of proteins. The sturdy conformations may mechanically jam the cell's protein-processing machinery, interfere with the breakdown of damaged or aggregated proteins, or otherwise stymie essential cell functions.

Based on their findings, Carrión-Vázquez and his collaborators suggest that extremely robust conformations are the primary cause of neurodegenerative disorders, so they could serve as ideal pharmacological targets as well as potential biomarkers for the early detection of disease. Their new approach opens the door to not only understanding the role of harmful protein accumulation in various conditions, but also developing effective therapeutic strategies.

**Hervás R, Oroz J, Galera-Prat A, Goñi O, Valbuena A, et al. (2012) Common Features at the Start of the Neurodegeneration Cascade. doi:10.1371/journal.pbio.1001335**

